# A Protein-Protein Interaction Map of the *Trypanosoma brucei* Paraflagellar Rod

**DOI:** 10.1371/journal.pone.0007685

**Published:** 2009-11-03

**Authors:** Sylvain Lacomble, Neil Portman, Keith Gull

**Affiliations:** Sir William Dunn School of Pathology and Oxford Centre for Integrative Systems Biology, University of Oxford, Oxford, United Kingdom; Temasek Life Sciences Laboratory, Singapore

## Abstract

We have conducted a protein interaction study of components within a specific sub-compartment of a eukaryotic flagellum. The trypanosome flagellum contains a para-crystalline extra-axonemal structure termed the paraflagellar rod (PFR) with around forty identified components. We have used a Gateway cloning approach coupled with yeast two-hybrid, RNAi and 2D DiGE to define a protein-protein interaction network taking place in this structure. We define two clusters of interactions; the first being characterised by two proteins with a shared domain which is not sufficient for maintaining the interaction. The other cohort is populated by eight proteins, a number of which possess a PFR domain and sub-populations of this network exhibit dependency relationships. Finally, we provide clues as to the structural organisation of the PFR at the molecular level. This multi-strand approach shows that protein interactome data can be generated for insoluble protein complexes.

## Introduction

Trypanosomatid protozoan parasites are the causative agents of a number of diseases responsible for the death of thousands of people in developing countries. There is currently little hope for a vaccine and existing treatment regimes are associated with high toxicity. All trypanosomes produce a single flagellum which is involved in numerous aspects of parasite biology including motility, cytokinesis, environment sensing, attachment to the host [1], [2], [3], [4], [5], [6] and in the case of the African trypanosome *Trypanosoma brucei*, correct generation and function of the flagellum is essential for the survival of the mammalian bloodstream stage [7]. The flagellum incorporates the canonical 9+2 microtubular eukaryotic axoneme and, as with the flagella of many species, has several additional lineage specific features. One of these additional features is an extra-axonemal para-crystalline structure termed the paraflagellar rod (PFR). The PFR of *T. brucei* has a complex subdomain organisation which includes proximal, intermediate, and distal domains as well as links to specific doublets of the axoneme and the flagellum attachment zone (FAZ) [8], [9]. In addition to its role in motility [10], [11], the PFR serves as a platform for metabolic and signaling enzymes [2], [12], [13] and is essential for the survival of the mammalian infective form of the parasite in the host bloodstream [14].

The eukaryotic flagellum is a widely conserved organelle and was a feature of the ancestor of all eukaryotes. The flagellum is implicated in an ever growing spectrum of human genetic disease [15] and is an attribute of many pathogenic organisms. It has been the subject of a number of recent proteomic characterisations in several model organisms and this analysis of components has also been extended to substructures of the axoneme such as the radial spokes and basal bodies [16], [17]. Our group has recently produced a *T. brucei* flagellar proteome [7] as well as a PFR proteome generated using comparative proteomics that consists of 30 high confidence proteins including 20 that were previously annotated in the genome as hypothetical [18]. Although these studies have improved our knowledge of the protein composition of the flagellum, the contribution of many of these newly identified components to structure and function remains to be determined.

A variety of functional genomics tools that have the potential to complement these large-scale proteomic assays of the flagellum are available. Yeast two-hybrid analyses have been extensively used to systematically investigate interactions of proteins in many model organisms [19], [20], [21], [22] at genome scale. Although local studies of flagellar functions have used the yeast two-hybrid approach to interrogate specific protein-protein interactions [23], [24], [25], [26], the utility of this technique for high throughput investigation has yet to be exploited for this organelle. A systematic interrogation of protein interactions in the flagellum is likely to provide valuable insight into its molecular architecture and functions. Another very powerful tool for the analysis of protein function in *T. brucei* is the ability to inducibly ablate protein expression by RNAi [27]. This has been done for individual proteins involved in a variety of cell processes as well as systematically as part of the Trypanofan project [28]. We have recently demonstrated that combining inducible RNAi with comparative proteomics techniques is a very powerful approach for the interrogation of dependent cohorts and sub-cohorts within complex protein structures [18]. We were able to identify an inter-dependent subset of proteins within the PFR cohort and propose a role for calcium signalling in the regulation of PFR adenylate kinases [18]. There is also evidence for either direct or indirect interactions between the major PFR components and calmodulin [13], strengthening the hypothesis for a major role for calcium in PFR function. We aim to provide evidence of interactions within the flagellum by a systematic yeast two-hybrid screen and to employ the above biochemical analyses to further elucidate the nature of the interactions.

Our PFR proteome forms a discreet subset of proteins within the flagellum and here we have used this as a training set for interrogating protein interactions and dependencies within the insoluble structural fraction of the flagellum. We have generated an open reading frame (ORF) library of 28 PFR proteins which is fully compatible with Gateway® cloning technology. We have used this library to perform a yeast two-hybrid screen and have identified novel protein-protein interactions within the PFR cohort. We have created a Gateway® compatible inducible RNAi vector based on p2T7-177 [29]–p2T7-177-GTW - and have successfully used this to ablate the expression of a number of PFR proteins. We have used this new vector in conjunction with biochemical and proteomics techniques to interrogate the nature of the interactions detected in the yeast two-hybrid screen and have identified new dependency networks and clues to the hierarchical nature of these interactions.

## Methods

### Cell Culture

Procyclic form *Trypanosoma brucei brucei* were cultured at 28°C in SDM 79 medium supplemented with 10% v/v foetal calf serum (Gibco) [30]. Cells were diluted as necessary to maintain the culture in log-phase and RNAi induction was achieved by the addition of doxycyclin to the culture medium to a final concentration of 1 µg ml^−1^.

### Vector Construction

The nucleotide sequence 5′-CTAGTGGGGACAACTTTGTACAAAAAAGTTGGCtcttgaggttccaggcttctcgagCCCAACTTTCTTGTACAAAGTTGTCCCCTTCGA-3′, containing AttB1, AttB2 and restriction enzyme recognition sites for XbaI, BamHI, HindIII and XhoI was inserted into p2T7-177 between the restriction sites for XbaI and ClaI such that the sites were destroyed. A BP reaction (Invitrogen) between this plasmid and pDNR223 was performed to obtain p2T7-177-GTW.

### ORFeome Construction

Open reading frames were amplified from *T. brucei* (strain 927) genomic DNA by PCR using primers containing AttB1 and AttB2 recognition sequences (Table S1) positioned relative to the ORF as previously described [31]. Purified ORF fragments were transferred to pDNR223 using BP clonase (Invitrogen) and from there to p2T7-177 GTW, pAD and pDB using LR clonase (Invitrogen). pDNR223, pAD and pDB were kind gifts of Prof Marc Vidal, Dana Farber Cancer Institute, Harvard University.

*Transfection.* 10–15 µg of purified linearised plasmid DNA was used to transfect 2.5 10^7^ of logarithmically growing 29∶13 procyclic form *T. brucei* [32] by electroporation (3×100 µs pulses of 1700 V at interval of 200 ms). Transfected cells were selected by the addition of 5 µg ml^−1^ Phleomycin to the growth medium.

### Preparation of Flagella, Western Blotting and DiGE Analysis

Cell fractionation, Western blotting and DiGE analyses were conducted as previously described [18].

### Immunofluorescence

Cells were settled onto glass slides and extracted by the addition of 1% Nonidet P-40 in PEME (100 mM PIPES, pH 6.9, 2 mM EGTA, 1 mM MgSO_4_, 0.1 mM EDTA). Extracted cells were fixed in methanol at −20°C and then labeled with BB2 [33] (α-Ty epitope). Labeling was visualized with 488 fluor-conjugated α-mouse IgG1 (Invitrogen). The slides were mounted in Vectashield mounting medium with 4′, 6′ - diamino-2-phenylindole (Vector Laboratories Inc) and examined on a Leica DM5500B.

### Transmission Electron Microscopy

For thin-section electron microscopy cells were fixed in culture by addition to the growth medium of glutaraldehyde to a final concentration of 2.5% (w/v). Cells were collected by centrifugation and fixed again in 4% (w/v) formaldehyde, 2% (w/v) glutaraldehyde in 100 mM sodium phosphate buffer, pH 7.0 before post-fixation with 1% (w/v) osmium tetroxide in 100 mM sodium phosphate buffer. After several washes, cells were en bloc stained with 1% (w/v) aqueous uranyl acetate before dehydration and embedding in epoxy resin. Sections were stained with aqueous uranyl acetate and Renyold's lead citrate, and viewed in an FEI Tecnai-F12 electron microscope operating at 80 kV.

### Yeast Two-Hybrid Assay

Yeast two-hybrid plasmids (pDB-ORF and pAD-ORF) were transformed in yeast cells (MaV103 (MATa) and MaV203 (MATα) respectively) and selected on medium lacking the leucine or tryptophan amino acid respectively. Auto-activation of each transformed MaV103 strain was tested on four selective media [34]. For the beta-galactosidase assay, patches of cells were plated on a nitrocellulose membrane placed onto a YPD plate and incubated overnight. Cells present on the nitrocellulose membrane were frozen in liquid nitrogen for 10 seconds and placed on Whatman paper filters pre-incubated with Z-buffer (8.52 g/L Na2HPO4, 5.5 g/L NaH2PO4, 0.75 g/L KCl and 0.12 g/L MgSO4) containing 0.18% (v/v) of 2-mercaptoethanol and 0.07% of bromo-4-chloro-3-indolyl B–D galactosidase (X-gal). Membranes were incubated at 37°C and photographed after 1 h, 4 h and 24 h. Pairs of interactions were examined by individually mating each MaV103-DB-ORF with MaV203-AD-ORF in 96 well plates containing 100 µL/well of YPD. Multi-well plates were incubated overnight at 30°C with shaking and then plated on selective medium lacking both leucine and tryptophan. After the generation of diploid cells, patches representing single potential interaction pairs were examined using the four yeast two-hybrid assays as previously described [34].

## Results

### A Yeast Two Hybrid Screen Identifies Eight Novel Interactions

We screened a number of PFR proteins identified in our comparative proteomics analysis [18] for interactions using a yeast two-hybrid assay. We successfully amplified 28 open reading frames by PCR and subsequently cloned them into the Gateway® compatible library vector pDNR223 using the BP clonase. This ‘ORFeome’ serves as a foundation for subsequent functional genomic analyses and we employed the Gateway® technology to transfer these open reading frames into the yeast two-hybrid plasmids pAD (prey) and pDB (bait).

**Figure 1:**
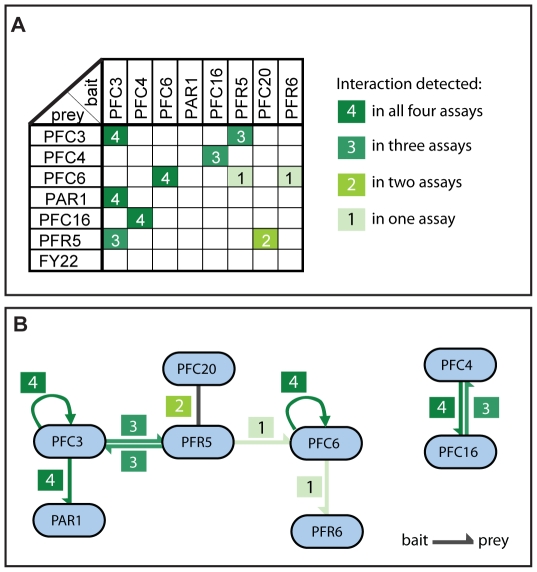
A yeast two-hybrid interaction map for the PFR. A. Summary table of all yeast two-hybrid interactions. For each pair, the number of assays in which an interaction was observed is shown. B. Cartoon representation of the yeast two-hybrid interactions described in A. The number of assays in which an interaction was detected is shown.

To identify protein-protein interactions in the PFR, we designed a matrix yeast two-hybrid assay by mating strains containing each bait to strains containing each prey and subsequently testing all 700 resulting pair-wise combinations (after removing auto-activators which are discussed later). Interactions were visualised using the one colorimetric and three auxotrophic assays described previously [34]. Resulting interactions were ranked according to the number of assays in which they were observed; 4 interactions were detected in all four assays, 3 interactions were detected in three assays, one interaction in two assays and two interactions in one assay. The interactions of PFC3-PFR5 and PFC4-PFC16 reciprocate in both bait and prey configurations and both PFC3 and PFC6 interact with themselves. All other interactions were seen in only one configuration (Figure S1). In total, 8 non-redundant interactions were detected in the yeast two-hybrid screen (Figure 1) and these can be divided into two clusters: PAR1-PFC3-PFR5-PFC20-PFC6-PFR6 and PFC4-PFC16. In the first of these clusters, PFC3, PFC6 and PFC20 all interact with PFR5 while PFC3 also interacts with PAR1.

Importantly, each of the bait constructs has been tested for self-activation in the four yeast two-hybrid assays (Figure S2). PFC2, PFC11 and PFC20 appear to be strong self-activators (in all four assays) preventing any further investigation of these in the bait configuration. PFC3 and PFR5 are weak auto-activators only in the colorimetric beta-galactosidase assay allowing the interactions of these proteins to be screened using the other three assays.

### A Gateway® Compatible RNAi Vector Facilitates a Functional Genomics Analysis

**Figure 2:**
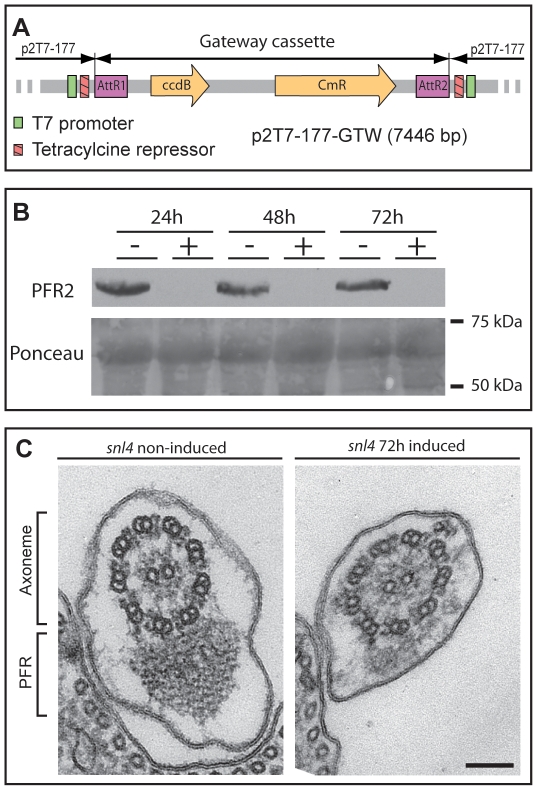
A Gateway® compatible vector for RNAi analysis in *T. brucei*. A. p2T7-177-GTW. The Gateway® cassette containing the chloramphenicol resistance gene and the toxic *ccdB* gene was inserted into the inducible expression site of the p2T7-177 plasmid. B. Western blotting analysis of whole cell lysates of *snl4* (procyclic form *T. brucei* containing p2T7-177-GTW-PFR2) over an RNAi induction time course. PFR2 protein (visualised with L8C4 antibody) is undetectable after 24 hours of RNAi induction. Ponceau stained membrane is shown as a loading control. C. Transmission electron micrographs of non-induced and 72 hour induced *snl4* flagellar cross-sections reveal the absence of a large part of the PFR structure. Bar 100 nm.

The availability of our PFR ORFeome in a Gateway® compatible format provides the opportunity to perform relatively high-throughput studies of PFR component function using genomics tools such as RNA interference. To facilitate this analysis, we modified the p2T7-177 inducible RNAi vector [29] to make it Gateway® compatible. We inserted the Gateway® cassette containing the *ccdB* toxic gene and the chloramphenicol resistance gene flanked by two attR recognition sequences necessary for LR recombination between the two tetracycline operators of p2T7-177 (Figure 2A). We named this plasmid p2T7-177-GTW and assessed its suitability by using it to generate a *T. brucei* procyclic form cell line (*snl4*) in which inducible RNAi is targeted against PFR2. We compared the phenotype of *snl4* with the previously described *snl2* PFR2 knock down mutant [35]. After RNAi induction, the level of PFR2 protein is reduced as revealed by Western blotting with the anti-PFR2 antibody L8C4 (Figure 2B). Defective motility is clearly apparent and cells are completely paralysed. At the structural level, EM analysis shows that the PFR structure is greatly reduced (Figure 2C) as seen in *snl2* [35], [36] demonstrating the utility of p2T7-177-GTW for future RNAi studies.

### Biochemical Analysis Provides Insight into the Nature of the PFC4-PFC16 Interaction

The interaction observed between PFC4 and PFC16 is particularly intriguing as we have previously shown that these otherwise dissimilar proteins share a small motif of 21 amino acids [18]. In order to interrogate whether this motif was responsible for this interaction, we constructed additional yeast two-hybrid vectors containing the domain of PFC4 and PFC16 alone in each bait/prey configuration and assayed the interactions of these with each of the full length open reading frames. No interaction was detected by any of the four yeast two-hybrid assays suggesting that this motif alone is not sufficient to maintain this protein interaction (data not shown).

**Figure 3:**
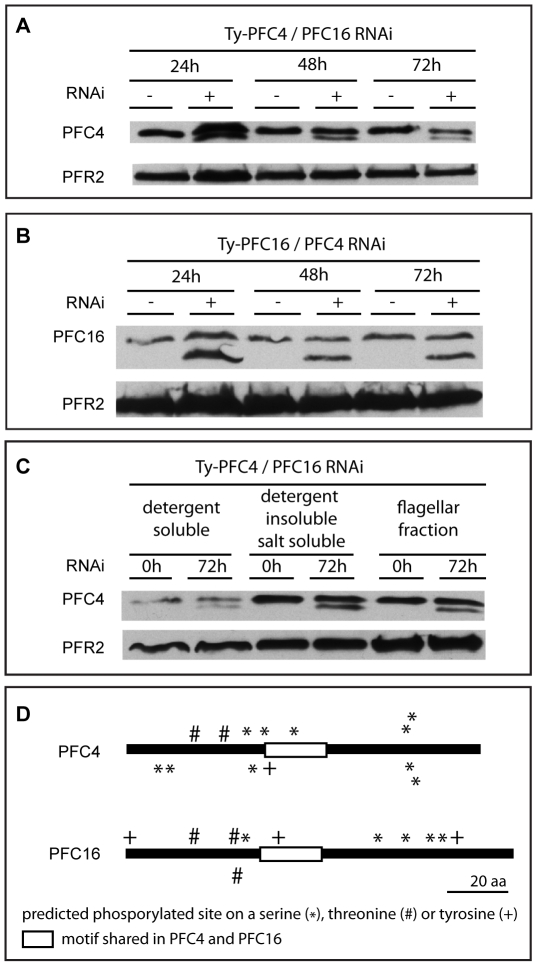
Analysis of PFC4 and PFC16 after induction of RNAi. A. Ty-PFC4 after RNAi ablation of PFC16. B. Ty-PFC16 after RNAi ablation of PFC4. In both cases a second band with higher mobility in the gel appears as soon as 24 hours after RNAi induction. C. Western blotting analysis of various cell fractions showing Ty-PFC4 after RNAi ablation of PFC16. Both bands are present in all cell fractions. Ty epitope tagged PFR proteins were detected using BB2 antibody. Loading control was performed using L8C4. D. Cartoon representation of predicted post-translational modification sites and conserved motifs for both PFC4 and PFC16.

We then asked whether ablation of one component had an effect on the other. We generated a cell line with a Ty epitope tagged copy of PFC4 [18] at one of the endogenous alleles and inducible RNAi (via p2T7-177-GTW) against PFC16. Whole cells lysates of non-induced, 24, 48 and 72 hours RNAi induced samples were compared by Western blotting using BB2 antibody which recognises the epitope tag. As expected, in the non-induced sample, a single band with apparent molecular weight consistent with the predicted molecular weight of Ty-PFC4 protein was observed. Interestingly, a second band with higher mobility in the gel was observed in samples made 24 hours after RNAi induction. This second band persisted until at least 72 hours after induction (Figure 3A). We performed a reciprocal analysis generating a cell line with Ty epitope tagged PFC16 in a PFC4 inducible RNAi background. Again, Western blotting of non-induced whole cell lysate with BB2 antibody revealed a single band consistent with the predicted molecular weight of Ty-PFC16 protein. Intriguingly, as before, a second band was observed after 24 h of RNAi induction that persisted until at least 72 h after RNAi induction (Figure 3B). Importantly, the RNAi induced bands in the two cell lines are of different apparent molecular weight and are therefore likely to be modified forms of the Ty epitope tagged protein in question and not an artefact of the RNAi. Western blotting of detergent and salt dissections of non-induced and induced Ty-PFC4/PFC16 RNAi cells revealed that both Ty-PFC4 bands are present in the same cell fractions (Figure 3C) suggesting that the localisation of this modified version of the protein is not altered.

In order to determine possible post-translational modifications of PFC4 and PFC16, we analysed the protein sequences using NetPhos [37] which suggests that PFC4 and PFC16 contain thirteen and eleven possible phosphorylation sites respectively. Further analysis using Phobius [38] Glycosylation predictor [39] and Myristoylator [40] predicts no glycosylation or myristoylation sites in either protein (Fig 3D).

### Proteomic Analysis Reveals Dependencies Supporting the Identified Interactions

**Figure 4:**
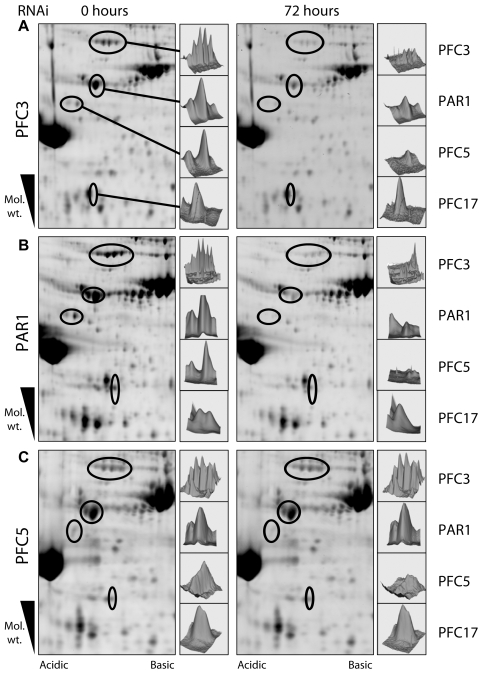
Two-dimensional DiGE analysis of PFC3, PAR1 and PFC5 non-induced and RNAi induced flagella. The gels were analyzed using Decyder software (GE Healthcare), which was used to generate three-dimensional representations of the spots that show a change in volume after induction. Reductions in volume were seen for spots corresponding to PFC3, PAR1, PFC5 and PFC17 in both PFC3 and PAR1 RNAi induced environments. Reduction in volume was seen only for spot corresponding to PFC5 following RNAi ablation of PFC5.

We have recently demonstrated the power of combining RNAi ablation of key proteins with comparative proteomics techniques to identify dependent sub-groups of proteins within the larger complex of the PFR [18]. This methodology can be used to detect changes in protein composition as a result of RNAi directed against specific components. We tested interactions detected in our yeast two-hybrid screen (PFC3/PAR1) using a combination of inducible RNAi, purification of flagella and 2D-DiGE analysis. Resulting spot patterns were matched using Decyder software to a reference 2D gel map of PFR proteins within the context of purified flagella. Samples prepared 72 hours after RNAi induction against PFC3 showed a number of spots with a twofold or greater decrease in volume when compared to non-induced samples (consistent with criteria applied previously– [18]) (Figure 4A). As expected a group of five spots of approximately 80 kDa that correspond to PFC3 showed two- to four-fold reduction in volume (3.8, 3.7, 3.1, 2.8 and 2.3-fold reductions). Interestingly the volume of a spot of approximately 65 kDa that corresponds to PAR1 also showed a 2.1-fold decrease. This result shows that PAR1 is not stably incorporated into the structure of the flagellum in the absence of PFC3 protein and supports the interaction of these two proteins observed in our yeast two-hybrid screen. Greater than twofold reductions in spot volumes following PFC3 RNAi were also observed for three other spots. These correspond to PFC5 (two spots–2.1-fold and 2.7-fold reductions in volume) and PFC17 (one spot–2.1-fold reduction in volume). PFC3 or PAR1 interactions with either PFC5 or PFC17 were not observed by yeast two-hybrid analysis. Reciprocal experiments showed that RNAi induction for 72 hours against PAR1 phenocopies the PFC3 result with spot volume reductions for PAR1 (3.7-fold reduction), PFC3 (3.1, 4.9, 4.5, 3.2 and 2.2-fold reductions), PFC5 (2.1 and 2.7-fold reductions) and PFC17 (2.1-fold reduction) (Figure 4B). RNAi against PFC5, however, only resulted in a reduction in volume of the two spots corresponding to PFC5 itself (1.5 and 2.0 -fold reductions) (Figure 4C). Taken together these data suggest a hierarchical dependency sub-network whereby PAR1 and PFC3 are both necessary for the incorporation of all four proteins into the PFR. RNAi/DiGE analyses were also carried out on the potential hub interactor, PFR5 but no protein composition phenotype, other than ablation of the RNAi target protein, were observed (data not shown).

### Ablation of the PFC3/PAR1 Complex Does Not Affect Structural Morphology of the PFR

**Figure 5:**
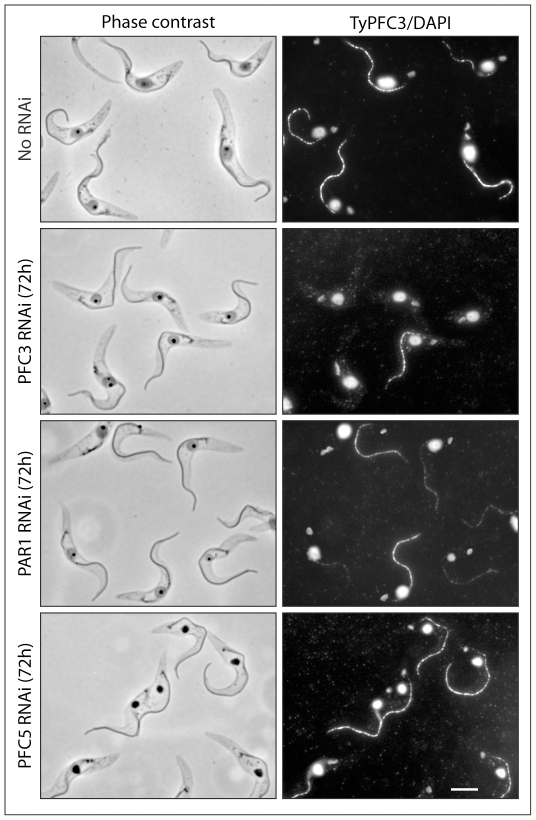
Immunofluorescence light microscopy of detergent extracted cells. BB2 antibody was used to detect Ty-PFC3 (flagellar signal) in PFC3, PAR1 and PFC5 RNAi environments (72 hours induction). Without RNAi induction, Ty-PFC3 is present in all flagella. After RNAi against PFC3 or PAR1, the majority of flagella no longer stain for Ty-PFC3 (it is likely that flagella that do stain represent those present prior to RNAi induction). After RNAi against PFC5, all flagella retain Ty-PFC3. DNA is visualised with DAPI. Bar–5 µm.

**Figure 6:**
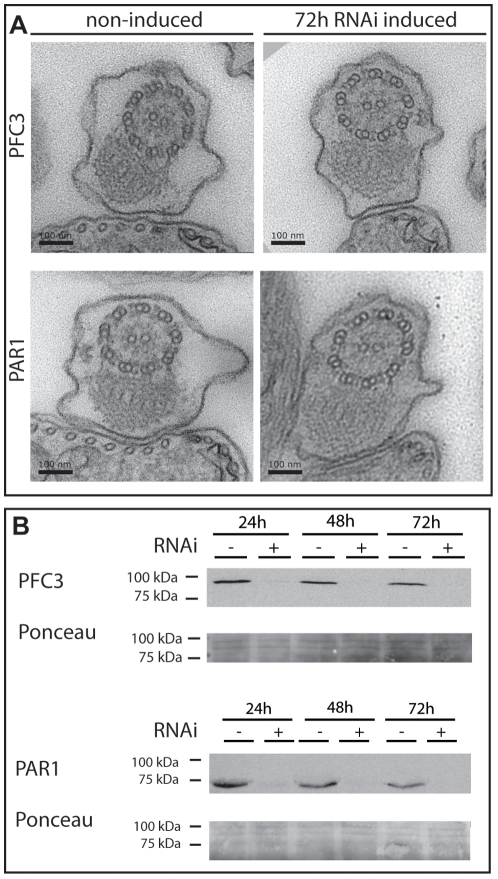
Analysis of PFC3 and PAR1 after induction of RNAi. A. Thin-section electron microscopy of whole cells reveals no detectable structural change in the PFR organisation after RNAi ablation of either PFC3 or PAR1. B. Western blotting analysis shows ablation of both PFC3 and PAR1 protein levels by 24 hours after induction of RNAi. Ty epitope tagged PFR proteins were detected using BB2 antibody. Ponceau stained membranes show loading of protein samples.

Using immunofluorescence light microscopy of detergent extracted cells, we analysed cell lines containing a Ty epitope tagged copy of PFC3 at one of the endogenous alleles (18) and inducible RNAi against PFC3, PAR1 or PFC5 with the BB2 antibody. Comparison of cells 72 hours after RNAi induction with non-induced controls showed no gross morphological changes (Figure 5). In addition, consistent with our previous results, PFC3 signal was absent in the majority of cells following PFC3 RNAi and was greatly reduced following PAR1 RNAi. Furthermore, RNAi against PFC5 did not affect PFC3 localisation. In order to determine if loss of the PFC3/PAR1 complex has any effect on the structure of the PFR, we analysed PFC3 and PAR1 RNAi mutants 72 hours after RNAi induction by thin section electron microscopy. To enable us to determine efficacy of the RNAi induction, we used cell lines containing an allele of the RNAi target tagged with the Ty epitope at the endogenous locus. Interestingly, despite the loss of two relatively high abundance proteins from the PFR (Figure 4), no observable changes could be detected in the PFR structure (Figure 6A). Western blotting analysis of samples taken from the cultures prior to fixation confirmed that the RNAi induction was successful as target proteins were ablated (Figure 6B).

## Discussion

Studies in *T. brucei* benefit from the availability of a completed and well annotated genome [41] as well as a wealth of functional genomics tools. This organism is an excellent model for the study of many eukaryotic cell processes, such as flagellar function, as well as being an important parasite in its own right. The almost total lack of introns in the genome and the poly-cistronic method of transcription employed by trypanosomatids greatly facilitate the prediction of open reading frames and thus aid in proteomic and functional genomics studies such as mass spectrometry, RNAi, overexpression and epitope tagging.

Historically, large scale forward genetic analyses have met with limited success in *T. brucei* [42], [43], and hence the need for systematic phenotype analysis remains paramount. To this end we have exploited cutting edge high-throughput Gateway® cloning strategies to produce an ORFeome library of PFR components. This ORFeome constitutes a platform for functional genomics studies to investigate protein interactions, sub-cellular localisation and mutant analyses.

We have developed a new vector utilising the well characterised RNAi reverse genetics techniques available for *T. brucei* that is fully compatible with the Gateway® technology. We have demonstrated the utility of this vector by ablating five distinct PFR proteins including PFR2 and in this last case have reproduced a phenotype consistent with the well characterised *snl2* mutant cell line. Taken together, these tools constitute a first step towards a high throughput functional genomics system in *T. brucei* that we have used here in conjunction with yeast two-hybrid and proteomics techniques to generate a protein-protein interaction network for the paraflagellar rod.

We have identified eight novel protein-protein interactions within the PFR using a yeast two hybrid screen of 28 PFR components. Our subsequent reverse genetics analysis of the protein set provided supporting evidence for a number of these interactions and also identified several novel protein dependencies not detected in our initial screen. It is perhaps surprising that, given the central role of PFR1 and PFR2 in the PFR, no interactions were detected between these two proteins and any of the others in this study. Yeast two-hybrid analyses typically suffer from a high frequency of false negative results and this may be a particular problem when dealing with very insoluble proteins such as PFR components. There is also the distinct possibility that many interactions within the PFR do not occur on a one-to-one basis. It may be that PFR1 and PFR2 must be part of a complex in order to interact with other PFR proteins or that less abundant components interact with PFR1 or PFR2 only as cohorts. Another possibility is that a chaperone-assisted folding process that is not present in yeast is required for some PFR component interactions. Finally, there is also the very likely possibility that we have not yet identified all of the PFR components and may be missing key mediators between PFR1/2 and the other PFR proteins. All of the interactions and dependencies that we identified fall into two clusters. The smaller cluster involves two proteins that share a motif, and the larger cluster involves eight proteins (one fifth of all identified PFR proteins) of which several contain a PFR domain.

The smaller cluster consists of PFC4 and PFC16 which show consistent interactions with each other in all yeast two-hybrid assays in both bait and prey configurations. We previously showed that PFC4 and PFC16 share a 21 amino acid motif [18], although this domain is not sufficient for maintaining the yeast two-hybrid interaction. RNAi/epitope tagging analyses reveal an intriguing relationship between these two proteins in that absence of one results in an additional higher mobility band for the other by Western blotting. We cannot currently exclude a role for degradation in this process; however the direct interaction of these two proteins suggests that the additional bands are the result of differential post-translational modifications although we have so far been unable to determine the nature of these modifications.

The larger cluster has two main hub interacting proteins, PFC3 and PFR5 which interact directly with each other. PFR5 interacts with PFC20 and possibly also with PFC6, although the evidence for this second interaction is weaker than that observed for other interactions in this screen. PFC6 interacts with itself and may also interact with PFR6, although again, the evidence for this latter interaction is relatively weak. It is interesting to note that PFC6 may interact with two proteins (PFR5, PFR6) that contain a PFR domain as well as interacting with itself. Similarly, the hub protein PFC3 interacts with two PFR domain containing proteins (PAR1, PFR5) as well as interacting with itself. We went on to investigate a number of these interactions by RNAi/DiGE analysis which has the power to provide context to the yeast two-hybrid data. While yeast two-hybrid analysis showed an interaction between PFC3 and PAR1, RNAi/DiGE provided an insight into the nature of this interaction in that PFC3 and PAR1 are mutually dependent on each other for correct assembly into the PFR structure. RNAi/DiGE analyses also reveal dependency relationships not detected in the yeast two-hybrid screen with incorporation of both PFC5 and PFC17 being dependent upon the presence of PFC3/PAR1. RNAi ablation of PFC5 does not affect the assembly of PFC3, PAR1 or PFC17 which suggests a hierarchical directionality to this dependency network.

One surprising, and likely significant result from our analysis is that a successful ablation by RNAi of the PFC3/PAR1 network results in no gross changes in the morphology of the PFR polymeric structure. This may represent evidence that the three major zones of the PFR and their distinct morphologies are heavily reliant for their formation on only a relatively few structural proteins, perhaps PFR1 and PFR2 (the only proteins that have been shown to localise to the PFR whose ablation results in a failure of PFR assembly). A picture then emerges of these few structural proteins being responsible for the major PFR lattice structure onto which is built a cohort of numerous other proteins with domain architectures suggesting a role in metabolism, signalling and regulation [2], [13], [18]. Taken together this supports the role of the PFR as an extra-axonemal structure critically involved in trypanosome sensory, motility and signalling cell biology [2], [10], [11], [13], [18]. It seems unlikely that abundant proteins such as PFC3 and PAR1, that are important enough to be conserved in the genomes of every sequenced trypanosomatid, lack a function. In these terms we will have to await the development of more sensitive phenotype tests before the function of these network proteins emerges.

**Figure 7:**
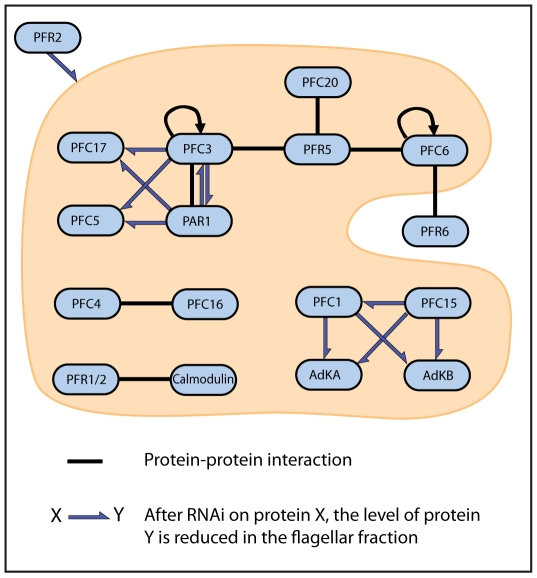
Summary of protein-protein interactions and dependencies of PFR components. Interactions drawn from this study and available literature [13], [18]; black bars represent interactions observed by yeast two-hybrid or affinity pulldown. Gray arrows represent dependencies detected by combined RNAi/comparative proteomics techniques.

In this study, we have developed a number of genomics tools for *T. brucei* and have used these to undertake the first large protein-protein interaction study of the PFR. Using a combination of technologies, we have identified eight novel protein-protein interactions and five protein dependencies. By combining the information presented in this study with the interactions and dependencies previously identified, we can summarise the current state of our knowledge of the PFR protein network (Fig. 7) to provide a framework for the continuing effort to elucidate the functions of this iconic structure. The position of PFR2 in the scheme represents the fact that many of the proteins included in this study were identified due to their dependence on PFR2, either directly or indirectly, for incorporation into the PFR. It is likely that PFR1 will occupy a similar position in such a network although there are currently no data available on protein components of the PFR that specifically depend on the presence of PFR1. A number of studies in recent years have provided insight into the PFR as a platform for regulatory and metabolic functions. This study supports the notion of a dynamic structure with a complex hierarchy of interacting and inter-dependent components [12], [13], [18].

## Supporting Information

Figure S1 Yeast 2-Hybrid screen. A. Summary table describing the interactions detected in the PFR screen with the associated protein-protein interaction network. B. The preys PFC1, PFR2 and PFC3 have been tested again all baits. The prey PFC3 interacts with both PFC3 and PFR5 baits in 4 and 3 assays respectively. C. PFC4 prey interacts with PFC16 baits in 3 assays and PFC6 prey interacts with itself (4 assays), with PFR5 and PFR6 (both in one assay).
(0.29 MB PDF)

Figure S2 Auto-activation assay of PFR baits. A. Cartoon illustrating that some ORFs fused with the Gal4 DNA binding domain have the ability to activate the transcription of the reporter genes.This behaviour has to be tested to decrease the false positive rate. B. The diagram shows the position of each DB-ORF for this assay. C. Readout of the four autoactivation assays (b-galactosidase assay, auxotrophic media lacking histidine and complemented with 20 and 60 mM 3AT and media lacking the uracil amino acid. PFC11, PFC2, PFC20 are strong auto-activators in all assays. PFC3 and PFR5 are weak auto-activators.
(0.06 MB PDF)

Table S1 PCR primers used to generate the ORFeome. AttB1 and AttB2 recognition sequences are shown in lower case.
(0.01 MB PDF)

## References

[1] Oberholzer M, Bregy P, Marti G, Minca M, Peier M (2007). Trypanosomes and mammalian sperm: one of a kind?. Trends Parasitol.

[2] Oberholzer M, Marti G, Baresic M, Kunz S, Hemphill A (2007). The Trypanosoma brucei cAMP phosphodiesterases TbrPDEB1 and TbrPDEB2: flagellar enzymes that are essential for parasite virulence.. FASEB J.

[3] Ogbadoyi EO, Robinson DR, Gull K (2003). A high-order trans-membrane structural linkage is responsible for mitochondrial genome positioning and segregation by flagellar basal bodies in trypanosomes.. Mol Biol Cell.

[4] Robinson DR, Gull K (1991). Basal body movements as a mechanism for mitochondrial genome segregation in the trypanosome cell cycle.. Nature.

[5] Beattie P, Gull K (1997). Cytoskeletal architecture and components involved in the attachment of Trypanosoma congolense epimastigotes.. Parasitology.

[6] Vickerman K (1973). The mode of attachment of Trypanosoma vivax in the proboscis of the tsetse fly Glossina fuscipes: an ultrastructural study of the epimastigote stage of the trypanosome.. J Protozool.

[7] Broadhead R, Dawe HR, Farr H, Griffiths S, Hart SR (2006). Flagellar motility is required for the viability of the bloodstream trypanosome.. Nature.

[8] Bastin P, Matthews KR, Gull K (1996). The paraflagellar rod of kinetoplastida: solved and unsolved questions.. Parasitol Today.

[9] Maga JA, LeBowitz JH (1999). Unravelling the kinetoplastid paraflagellar rod.. Trends Cell Biol.

[10] Santrich C, Moore L, Sherwin T, Bastin P, Brokaw C (1997). A motility function for the paraflagellar rod of Leishmania parasites revealed by PFR-2 gene knockouts.. Mol Biochem Parasitol.

[11] Bastin P, Sherwin T, Gull K (1998). Paraflagellar rod is vital for trypanosome motility.. Nature.

[12] Pullen TJ, Ginger ML, Gaskell SJ, Gull K (2004). Protein targeting of an unusual, evolutionarily conserved adenylate kinase to a eukaryotic flagellum.. Mol Biol Cell.

[13] Ridgley E, Webster P, Patton C, Ruben L (2000). Calmodulin-binding properties of the paraflagellar rod complex from Trypanosoma brucei.. Mol Biochem Parasitol.

[14] Griffiths S, Portman N, Taylor PR, Gordon S, Ginger ML (2007). RNA interference mutant induction in vivo demonstrates the essential nature of trypanosome flagellar function during mammalian infection.. Eukaryot Cell.

[15] Gerdes JM, Davis EE, Katsanis N (2009). The vertebrate primary cilium in development, homeostasis, and disease.. Cell.

[16] Yang P, Diener DR, Yang C, Kohno T, Pazour GJ (2006). Radial spoke proteins of Chlamydomonas flagella.. J Cell Sci.

[17] Kilburn CL, Pearson CG, Romijn EP, Meehl JB, Giddings TH (2007). New Tetrahymena basal body protein components identify basal body domain structure.. J Cell Biol.

[18] Portman N, Lacomble S, Thomas B, McKean PG, Gull K (2009). Combining RNA interference mutants and comparative proteomics to identify protein components and dependences in a eukaryotic flagellum.. J Biol Chem.

[19] Rual JF, Venkatesan K, Hao T, Hirozane-Kishikawa T, Dricot A (2005). Towards a proteome-scale map of the human protein-protein interaction network.. Nature.

[20] Li S, Armstrong CM, Bertin N, Ge H, Milstein S (2004). A map of the interactome network of the metazoan C. elegans.. Science.

[21] LaCount DJ, Vignali M, Chettier R, Phansalkar A, Bell R (2005). A protein interaction network of the malaria parasite Plasmodium falciparum.. Nature.

[22] Uetz P, Giot L, Cagney G, Mansfield TA, Judson RS (2000). A comprehensive analysis of protein-protein interactions in Saccharomyces cerevisiae.. Nature.

[23] Ahmed NT, Gao C, Lucker BF, Cole DG, Mitchell DR (2008). ODA16 aids axonemal outer row dynein assembly through an interaction with the intraflagellar transport machinery.. J Cell Biol.

[24] Tam LW, Wilson NF, Lefebvre PA (2007). A CDK-related kinase regulates the length and assembly of flagella in Chlamydomonas.. J Cell Biol.

[25] Lucker BF, Behal RH, Qin H, Siron LC, Taggart WD (2005). Characterization of the intraflagellar transport complex B core: direct interaction of the IFT81 and IFT74/72 subunits.. J Biol Chem.

[26] Zhang Z, Jones BH, Tang W, Moss SB, Wei Z (2005). Dissecting the axoneme interactome: the mammalian orthologue of Chlamydomonas PF6 interacts with sperm-associated antigen 6, the mammalian orthologue of Chlamydomonas PF16.. Mol Cell Proteomics.

[27] Motyka SA, Englund PT (2004). RNA interference for analysis of gene function in trypanosomatids.. Curr Opin Microbiol.

[28] Subramaniam C, Veazey P, Redmond S, Hayes-Sinclair J, Chambers E (2006). Chromosome-wide analysis of gene function by RNA interference in the african trypanosome.. Eukaryot Cell.

[29] Wickstead B, Ersfeld K, Gull K (2002). Targeting of a tetracycline-inducible expression system to the transcriptionally silent minichromosomes of Trypanosoma brucei.. Mol Biochem Parasitol.

[30] Brun R, Jenni L (1977). A new semi-defined medium for Trypanosoma brucei sspp.. Acta Trop.

[31] Rual JF, Hirozane-Kishikawa T, Hao T, Bertin N, Li S (2004). Human ORFeome version 1.1: a platform for reverse proteomics.. Genome Res.

[32] Wirtz E, Leal S, Ochatt C, Cross GA (1999). A tightly regulated inducible expression system for conditional gene knock-outs and dominant-negative genetics in Trypanosoma brucei.. Mol Biochem Parasitol.

[33] Bastin P, Bagherzadeh Z, Matthews KR, Gull K (1996). A novel epitope tag system to study protein targeting and organelle biogenesis in Trypanosoma brucei.. Mol Biochem Parasitol.

[34] Walhout AJ, Vidal M (2001). High-throughput yeast two-hybrid assays for large-scale protein interaction mapping.. Methods.

[35] Bastin P, Ellis K, Kohl L, Gull K (2000). Flagellum ontogeny in trypanosomes studied via an inherited and regulated RNA interference system.. J Cell Sci.

[36] Bastin P, Pullen TJ, Sherwin T, Gull K (1999). Protein transport and flagellum assembly dynamics revealed by analysis of the paralysed trypanosome mutant snl-1.. J Cell Sci.

[37] Blom N, Gammeltoft S, Brunak S (1999). Sequence and structure-based prediction of eukaryotic protein phosphorylation sites.. J Mol Biol.

[38] Kall L, Krogh A, Sonnhammer EL (2004). A combined transmembrane topology and signal peptide prediction method.. J Mol Biol.

[39] Hamby SE, Hirst JD (2008). Prediction of glycosylation sites using random forests.. BMC Bioinformatics.

[40] Bologna G, Yvon C, Duvaud S, Veuthey AL (2004). N-Terminal myristoylation predictions by ensembles of neural networks.. Proteomics.

[41] Berriman M, Ghedin E, Hertz-Fowler C, Blandin G, Renauld H (2005). The genome of the African trypanosome Trypanosoma brucei.. Science.

[42] Monnerat S, Clucas C, Brown E, Mottram JC, Hammarton TC (2009). Searching for novel cell cycle regulators in Trypanosoma brucei with an RNA interference screen.. BMC Res Notes.

[43] Morris JC, Wang Z, Drew ME, Englund PT (2002). Glycolysis modulates trypanosome glycoprotein expression as revealed by an RNAi library.. EMBO J.

