# Supplementary material for: A Protein-Protein Interaction Map of the Trypanosoma brucei Paraflagellar Rod
Source: PLoS One. 2009 Nov 3;4(11):e7685. doi: 10.1371/journal.pone.0007685 (PMC2766642; doi:10.1371/journal.pone.0007685)
Supplement: Figure S1 — Yeast 2-Hybrid screen. A. Summary table describing the interactions detected in the PFR screen with the associated protein-protein interaction network. B. The preys PFC1, PFR2 and PFC3 have been tested again all baits. The prey PFC3 interacts with both PFC3 and PFR5 baits in 4 and 3 assays respectively. C. PFC4 prey interacts with PFC16 baits in 3 assays and PFC6 prey interacts with itself (4 assays), with PFR5 and PFR6 (both in one assay). (0.29 MB PDF) [file pone.0007685.s001.pdf]

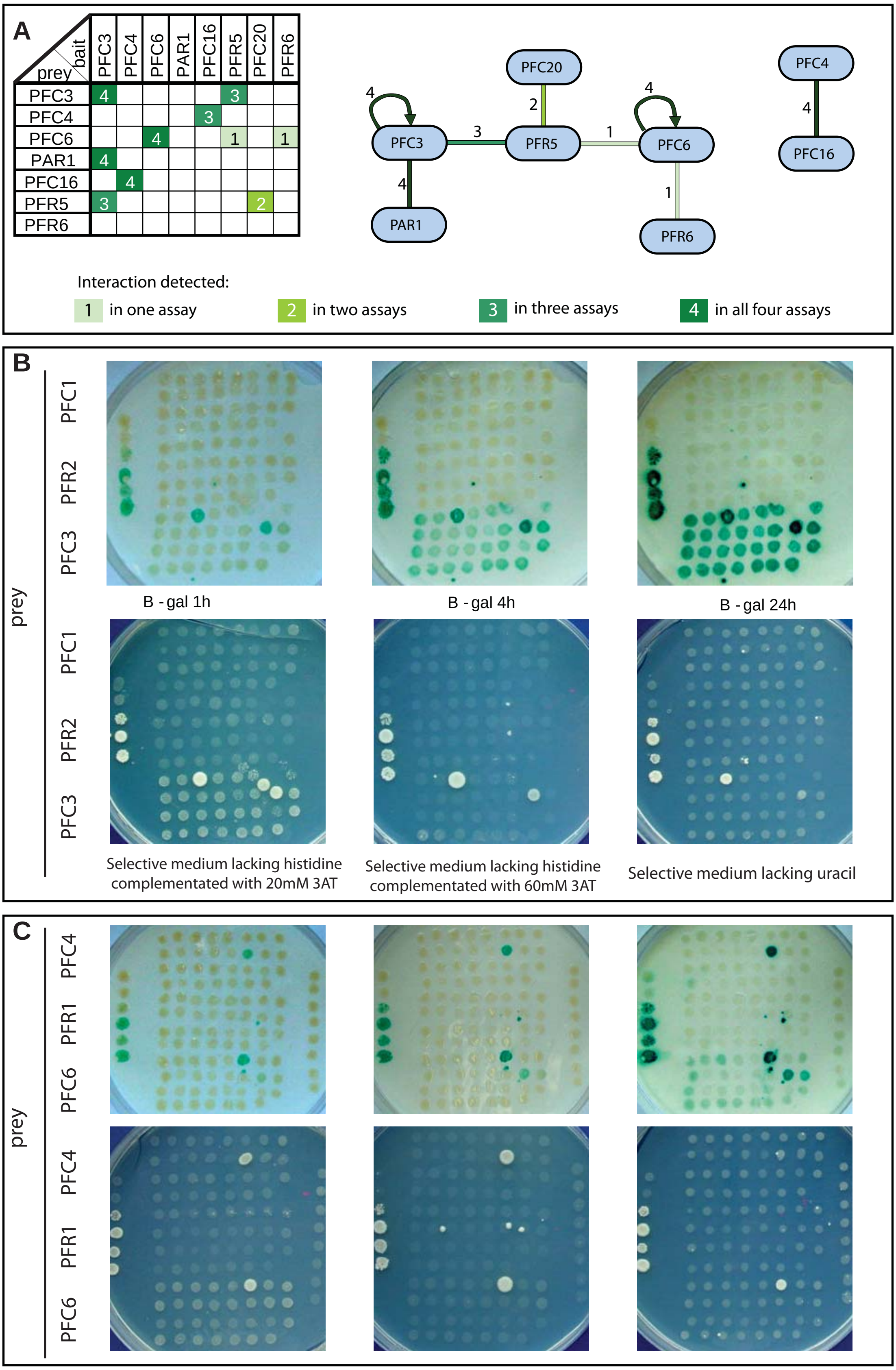

Supplemental figure 1: Y2H screen. A. Summary table describing the interactions detected in the PFR screen with the associated protein-protein interaction network. B. The preys PFC1, PFR2 and PFC3 have been tested again all baits. The prey PFC3 interacts with both PFC3 and PFR5 baits in 4 and 3 assays respectively. C. PFC4 prey interacts with PFC16 baits in 3 assays and PFC6 prey interacts with itself (4 assays), with PFR5 and PFR6 (both in one assay).

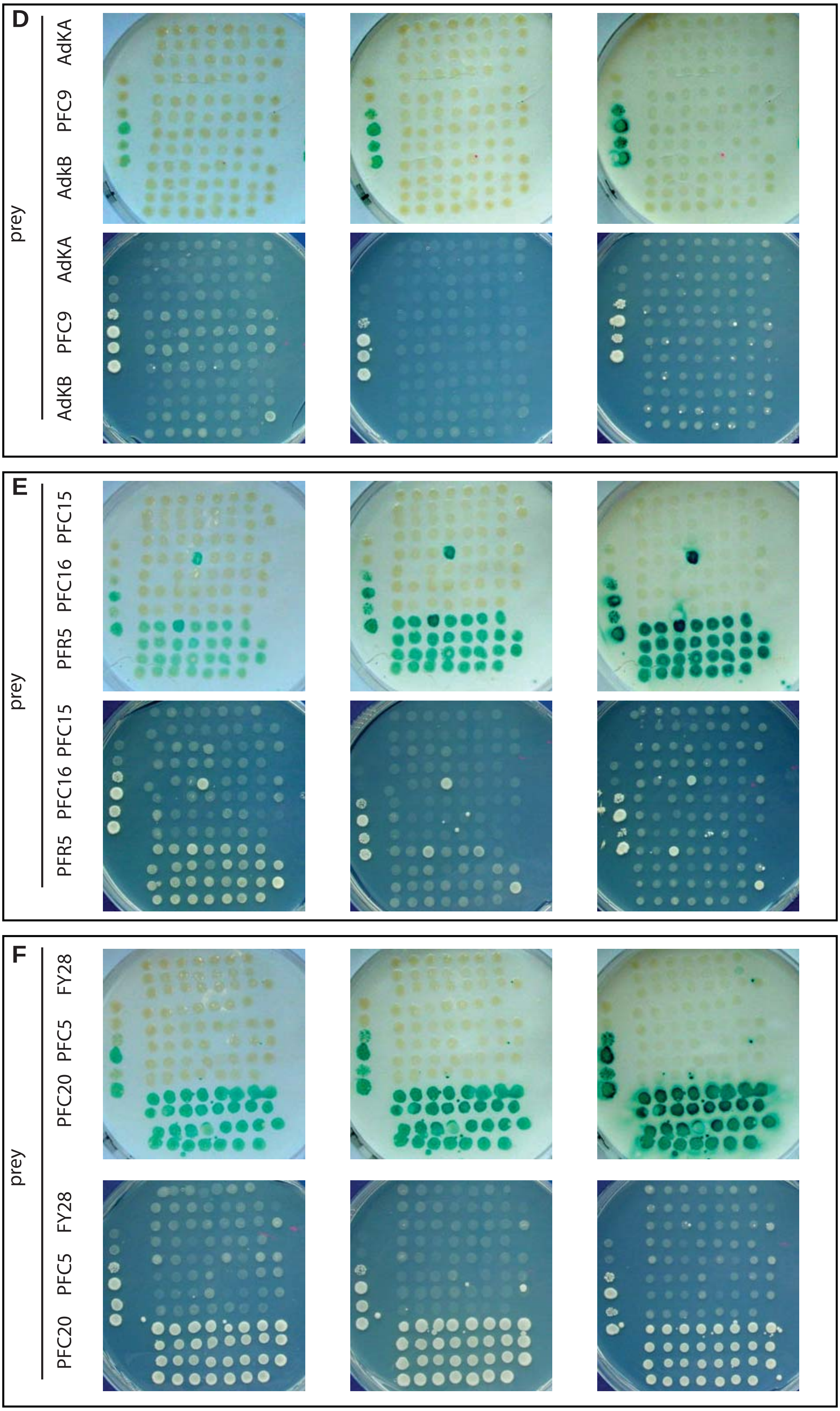

Supplemental figure 1: Y2H screen. D. No interaction has been detected for the preys AdKA, PFC9 and AdKB. E. The prey PFC16 interacts with PFC4 (in 4 assays). PFR5 interacts with PFC3 in 3 assays as well as with PFC20 (2 assays). F. No interaction was detected for all FY28, PFC20 and PFC5 preys in any assays.

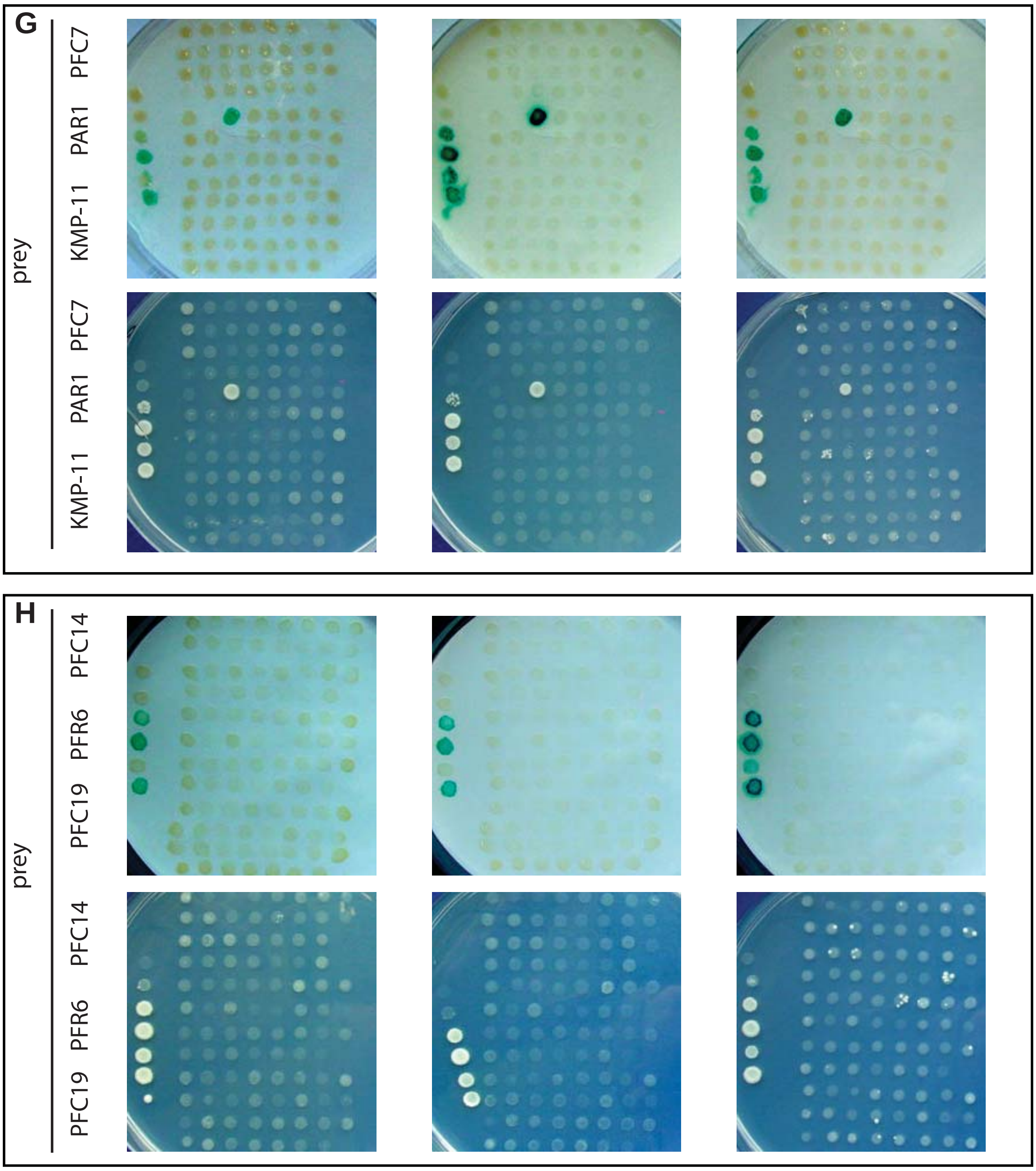

Supplemental figure 1: <u>Y2H screen.</u> G. PAR1 interacts in all assays with PFC3. H. No interaction was detected for the preys PFC14, PFR6 and PFC19 against all baits.
