# Supplementary material for: A Protein-Protein Interaction Map of the Trypanosoma brucei Paraflagellar Rod
Source: PLoS One. 2009 Nov 3;4(11):e7685. doi: 10.1371/journal.pone.0007685 (PMC2766642; doi:10.1371/journal.pone.0007685)
Supplement: Figure S2 — Auto-activation assay of PFR baits. A. Cartoon illustrating that some ORFs fused with the Gal4 DNA binding domain have the ability to activate the transcription of the reporter genes.This behaviour has to be tested to decrease the false positive rate. B. The diagram shows the position of each DB-ORF for this assay. C. Readout of the four autoactivation assays (b-galactosidase assay, auxotrophic media lacking histidine and complemented with 20 and 60 mM 3AT and media lacking the uracil amino acid. PFC11, PFC2, PFC20 are strong auto-activators in all assays. PFC3 and PFR5 are weak auto-activators. (0.06 MB PDF) [file pone.0007685.s002.pdf]

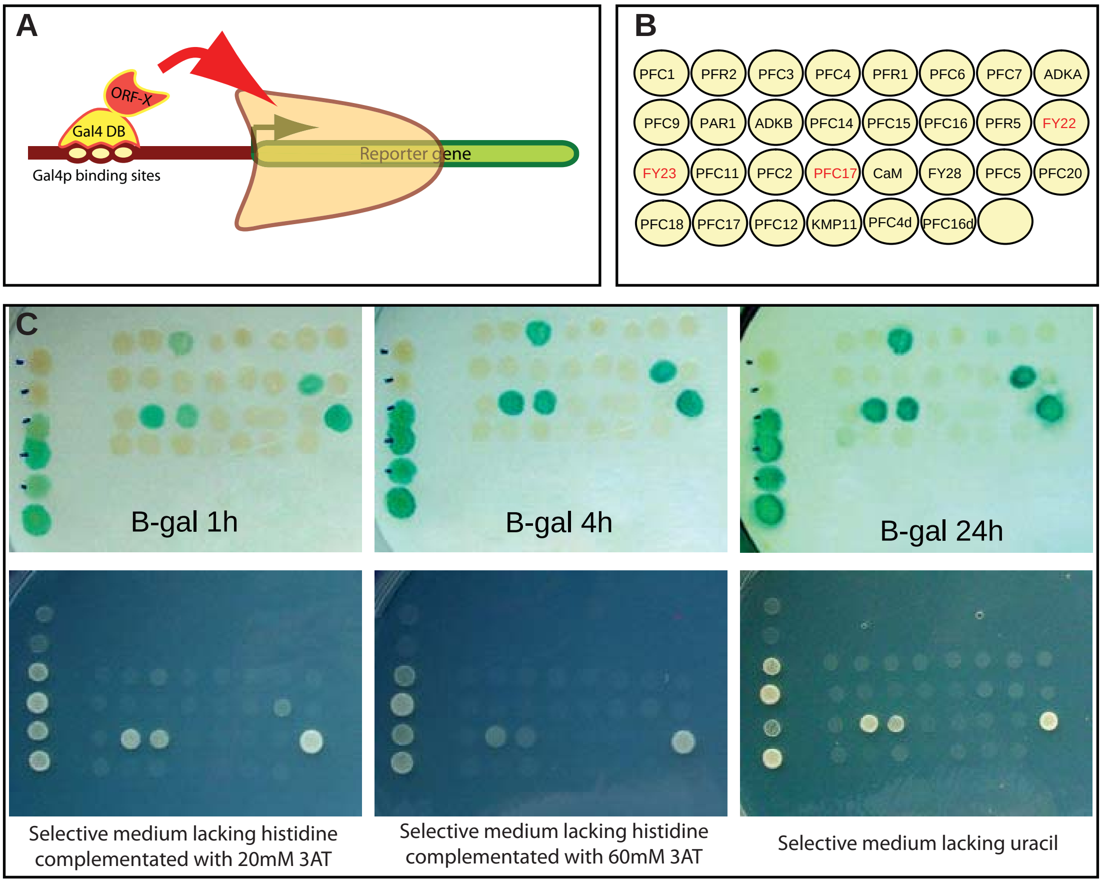

Supplemental Figure 2: Auto-activation assay of PFR baits. A. Cartoon illustrating that some ORFs fused with the Gal4 DNA binding domain have the ability to activate the transcription of the reporter genes.This behaviour has to be tested to decrease the false positive rate. B. The diagram shows the position of each DB-ORF for this assay. C. Read-out of the four autoactivation assays (β-galactosidase assay, auxotrophic media lacking histidine and complemented with 20 and 60 mM 3AT and media lacking the uracil amino acid. PFC11, PFC2, PFC20 are strong auto-activators in all assays. PFC3 and PFR5 are weak auto-activators.
