# Supplementary material for: A Protein-Protein Interaction Map of the Trypanosoma brucei Paraflagellar Rod
Source: PLoS One. 2009 Nov 3;4(11):e7685. doi: 10.1371/journal.pone.0007685 (PMC2766642; doi:10.1371/journal.pone.0007685)
Supplement: Table S1 — PCR primers used to generate the ORFeome. AttB1 and AttB2 recognition sequences are shown in lower case. (0.01 MB PDF) [file pone.0007685.s003.pdf]

Accession number	Gene name	Forward primer	Reverse primer
Tb927.8.6660	PFC1	ggggacaactttgtacaaaaaagttggcatgATGACGACGATGCAGAG	ggggacaactttgtacaagaaagttgggTAAGGTGCGAAGGCTG
Tb927.8.4970	PFR2	ggggacaactttgtacaaaaaagttggcatgATGAGCGGAAAGGAAGTT	ggggacaactttgtacaagaaagttgggTACTGAGTGATCTGCGG
Tb927.8.1550	PFC3	ggggacaactttgtacaaaaaagttggcatgATGTCGGCCACTTATCC	ggggacaactttgtacaagaaagttgggTATGCGCACTCCTCTAC
Tb927.6.4140	PFC4	ggggacaactttgtacaaaaaagttggcatgATGCCAAACCTCGTTGT	ggggacaactttgtacaagaaagttgggGAGCGAGTGAAATAAAACTTAATGC
Tb927.3.4290	PFR1	ggggacaactttgtacaaaaaagttggcatgGTTGACGATGCCACTG	ggggacaactttgtacaagaaagttgggGGTGCAGTGTTGTTTCC
Tb927.3.3770	PFC6	ggggacaactttgtacaaaaaagttggcatgATGAGTGCAACAGAGGC	ggggacaactttgtacaagaaagttgggTAGTACCTAGAGAACCGTGTC
Tb927.3.3750	PFC7	ggggacaactttgtacaaaaaagttggcatgTCGACTCAACTTATTTCCGGT	ggggacaactttgtacaagaaagttgggCGCAGCCACGAAAGAA
Tb927.2.5660	ADKA	ggggacaactttgtacaaaaaagttggcatgGCGGTACTGAGCGAAG	ggggacaactttgtacaagaaagttgggCGAAAGCGGTGCTTCT
Tb11.01.6510	PFC9	ggggacaactttgtacaaaaaagttggcatgATGTCTGCTGACCCCA	ggggacaactttgtacaagaaagttgggTGAGCCTCCGCATTTTC
Tb11.01.5100	PAR1	ggggacaactttgtacaaaaaagttggcatgATGATCGAGGTGCAACTTAAC	ggggacaactttgtacaagaaagttgggCGAATCGCCAACTCGA
Tb10.70.7330	ADKB	ggggacaactttgtacaaaaaagttggcatgGACCTTTCCGATAATAAGTTTGC	ggggacaactttgtacaagaaagttgggCGGCTCTCCACTGTTT
Tb10.6k15.0810	PFC14	ggggacaactttgtacaaaaaagttggcatgATGGAGAAAACACAACCGT	ggggacaactttgtacaagaaagttgggTACTCTTTCACAAGAGCCC
Tb10.61.1260	PFC15	ggggacaactttgtacaaaaaagttggcatgATGGCAAGTTCTACAGCAA	ggggacaactttgtacaagaaagttgggTACATCTCTGGGTTCTCCT
Tb10.26.0680	PFC16	ggggacaactttgtacaaaaaagttggcatgATGTCTCACTACCTTTTGGTCA	ggggacaactttgtacaagaaagttgggCAGCTATCCTCAGCAAGAAG
Tb927.2.4330	PFR5	ggggacaactttgtacaaaaaagttggcatgATGACGCAATCAGCTCAA	ggggacaactttgtacaagaaagttgggCAATCATCTTCATCACAGGGT
Tb927.7.6970	PFR6	ggggacaactttgtacaaaaaagttggcatgAAAAAGAGTGGTCATGAGGG	ggggacaactttgtacaagaaagttgggTATGAAGACGTTTTAGGCGC
Tb10.6k15.0140	PFC19	ggggacaactttgtacaaaaaagttggcatgGTTCAGGGCCACTTGT	ggggacaactttgtacaagaaagttgggTACGAAGCTTTCACTGAGG
Tb927.2.2160	PFC11	ggggacaactttgtacaaaaaagttggcatgATGACATCGGAGGAAGC	ggggacaactttgtacaagaaagttgggTAAATGTATTGCTCCAAATGCTCA
Tb927.8.3790	PFC2	ggggacaactttgtacaaaaaagttggcatgATGGATGCAGAAGAGAATTTCCT	ggggacaactttgtacaagaaagttgggTACGCACTGTTCGCAG
Tb11.01.3000	PFC17	ggggacaactttgtacaaaaaagttggcatgAAGAACAAACCCAGTAGTTATGC	ggggacaactttgtacaagaaagttgggCTTCTGTTTCTGCGCTCT
Tb11.01.4623	Calmodulin	ggggacaactttgtacaaaaaagttggcatgATGGCCGATCAACTCTC	ggggacaactttgtacaagaaagttgggTATTTGCTCATCATCATTTTCACGA
Tb927.2.3020		ggggacaactttgtacaaaaaagttggcatgATGTCTGATGCGTCCG	ggggacaactttgtacaagaaagttgggCACTTACCCTCCTCTCC
Tb927.7.1920	PFC5	ggggacaactttgtacaaaaaagttggcatgATGTTACAAGCGGCTGA	ggggacaactttgtacaagaaagttgggCGTCGATTAAACTTTTTCAGGTTC
Tb10.389.0100	PFC20	ggggacaactttgtacaaaaaagttggcATGGATCAATCATGCAACGAA	ggggacaactttgtacaagaaagttgggCGTGGGATCCGATACG
Tb10.6k15.1510	PFC18	ggggacaactttgtacaaaaaagttggcatgGACTGTGTGTATAACTCAGTGG	ggggacaactttgtacaagaaagttgggTACTGGCGAGCTAATGC
Tb11.01.3000	PFC17	ggggacaactttgtacaaaaaagttggcatgAAGAACAAACCCAGTAGTTATGC	ggggacaactttgtacaagaaagttgggCTTCTGTTTCTGCGCTCT
Tb11.02.2350	PFC12	ggggacaactttgtacaaaaaagttggcATGGCGAAAAAGGGTAAAAAGA	ggggacaactttgtacaagaaagttgggCAGTGTGAAGGCAACATATC
Tb927.6.4140	domain of PFC4	ggggacaactttgtacaaaaaagttggcTATTTTGTGCACAGCAAAAACC	ggggacaactttgtacaagaaagttgggTGGTCACAAAAGTGTTGACTTAATTCC
Tb10.26.0680	domain of PFC16	ggggacaactttgtacaaaaaagttggcAAGTTTGAGTACCTTCCCAAC	ggggacaactttgtacaagaaagttgggTGGTCGCAAAACTGTCTGTC
Tb09.211.4513	KMP-11	ggggacaactttgtacaaaaaagttggcATGGCCACCACATACG	ggggacaactttgtacaagaaagttgggGGGAACTGGGCATTCT
